# Imaging-Based Artificial Intelligence in Vascular and Interventional Radiology: A Narrative Review

**DOI:** 10.1007/s10278-025-01803-0

**Published:** 2026-01-05

**Authors:** Haseeb Mukhtar, Ali Ganjizadeh, Ajay Misra, Jamie Kane, Ping Wang, Prabh G. Singh, Alaura Lemieux, Gaurav Baranwal, Sreenivasulu Kilari, Bradley J. Erickson, Sanjay Misra

**Affiliations:** 1 Department of Radiology, Mayo Clinic, Rochester, MN USA; 2 Vascular Interventional Radiology Translational Laboratory, Mayo Clinic, Rochester, MN USA; 3 Radiology Informatics Laboratory, Mayo Clinic, Rochester, MN USA

**Keywords:** Imaging, Machine learning, Interventional radiology

## Abstract

Artificial intelligence (AI) has shown remarkable success in diagnostic radiology through advanced pattern recognition capabilities, yet its application in vascular interventional radiology (VIR) remains limited due to smaller, more variable datasets. This review examines AI applications in VIR procedures that utilize imaging modalities as input across preprocedural, intraprocedural, and post-procedural stages. A comprehensive literature search across PubMed, Embase, and Web of Science identified studies employing AI models with direct patient care impact, categorized by imaging modality (CT, MRI, fluoroscopy/DSA, ultrasound, X-ray, and multimodal) and task type (segmentation, detection, prediction, and miscellaneous). AI demonstrated substantial promise across multiple VIR domains. Deep learning models achieved high Dice similarity coefficients (0.82–0.962) for anatomical structure segmentation including aortic dissections and abdominal aortic aneurysms. Detection tasks showed excellent performance with accuracies up to 95% for endoleak detection and AUCs reaching 0.97 for vessel stenosis identification. Prediction models frequently outperformed traditional clinical assessments, with AUCs exceeding 0.90 for outcomes after EVAR, TEVAR, TACE, and TARE procedures. Radiomics-based approaches combined with machine learning proved particularly effective for treatment response prediction and risk stratification. Despite challenges including limited dataset sizes, potential bias, and interpretability concerns, AI shows transformative potential in VIR. Continued clinician AI expert collaboration will be crucial for responsible deployment and optimization of patient care in interventional radiology.

**Supplementary Information:**

The online version contains supplementary material available at https://doi.org/10.1007/s10278-025-01803-0.

## Introduction

In recent years, artificial intelligence (AI) has made significant advancements in medicine, particularly in radiology [1, 2]. Unlike the qualitative judgments made by radiologists, AI excels in recognizing complex patterns in imaging data and making efficient quantitative assessments [1]. This capability has been enhanced by the incorporation of deep neural networks for medical image analysis. With the digitization of radiology and the abundance of data in diagnostic radiology, AI has found successful applications in this field, which thrives on data-rich environments for optimal training [3]. In contrast, the application of AI in interventional radiology (IR) and specifically vascular IR (VIR) has been relatively limited due to smaller and highly variable case data [3]. However, there is huge potential for AI in VIR as evidenced by the work of many. AI can assist not only with intraprocedural image interpretation but also with preprocedural tasks like patient selection and post-procedural tasks such as determining patient prognosis [4].

The objective of this review is to look at the progress made by AI within the domain of VIR with respect to the different imaging modalities used.

## Methodology

A comprehensive literature search was conducted on PubMed, Embase, and Web of Science by AG and HM. The search strings, constructed by AG and HM, are detailed in Sect. 1 of the Supplementary Material. Study selection and review were carried out by HM, AG, and AM. Articles were included if at least two of the three reviewers agreed on their inclusion. The manuscript was written collaboratively by all authors, with HM and AG leading the drafting process. The remaining authors contributed additional content and edits prior to the initial submission of the manuscript.

Original articles up until April 2025 were included if they were in English and utilized an AI model in any of the three main steps of vascular IR (VIR) procedures (pre-procedure, intra-procedure, and post-procedure) with a direct impact on patient care. Studies based on neurointerventions and interventional cardiology procedures were not included in this narrative review; some good examples for reviews already done within these fields would be the works of Abdollahifard et al. [5] and Rudnicka et al. [6], respectively.

The AI models were developed using various imaging modalities as input, with a focus on computed tomography (CT), fluoroscopy, and angiography (including digital subtraction angiography), magnetic resonance imaging (MRI), ultrasound, X-ray, and combinations of the above. The tasks of the AI models were classified into prognostication and prediction, detection, segmentation, and miscellaneous tasks such as registration and interpolation.

## Results

**Fig. 1:**
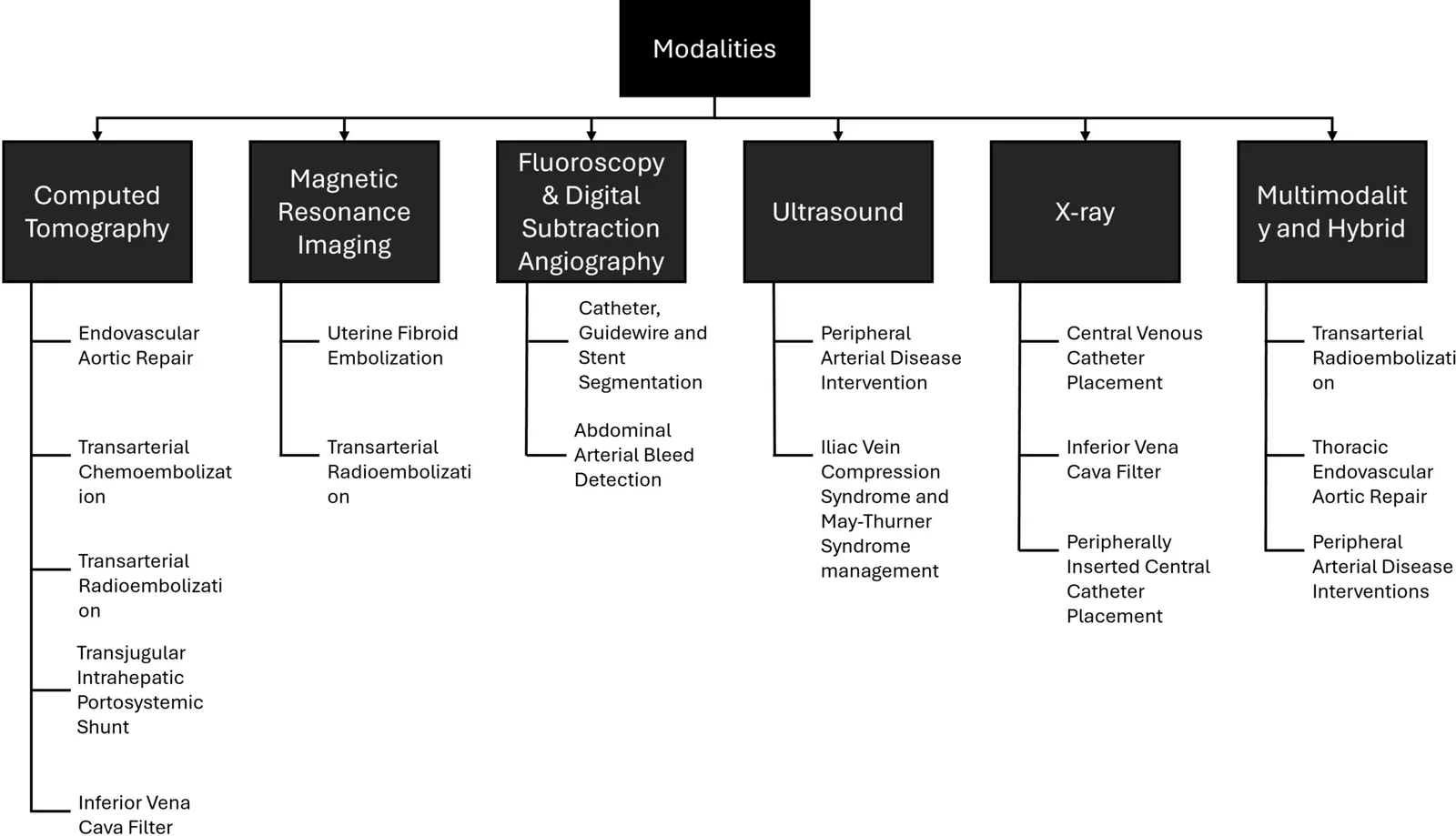
Different modalities and procedures studied by the AI models

This section is divided based on the imaging modality, with a subdivision based on the task of the AI model (Fig. 1). Additional information regarding the articles, including sample size/dataset, procedure involved, and short description, is mentioned in the supplementary data tables (Table E1-E7).

### Computed Tomography (CT)-Based Studies

#### Segmentation Tasks

Several studies focused on the segmentation of abdominal aortic aneurysm (AAA) sac, thrombus, and branch vessels using CT images. Patient-specific aortic dissection models after thoracic endovascular aortic repair (TEVAR) are necessary for making accurate measurements and hemodynamic analysis. However, a rapid and accurate technique for achieving that is unavailable. In addition, no general consensus for taking such measurements exists. A study proposed a deep learning (DL)-based multi-stage segmentation framework for type-B aortic dissection treated with TEVAR which achieved DSCs of 0.962, 0.921, 0.811, and 0.884 for true lumen, false lumen, thrombus, and branch vessels, respectively [7]. Another study used 3D CT images to automatically segment the thoracic aorta and pinpoint proximal landing zones together with providing measurements necessary for TEVAR planning. Achieving a mean DSC of 0.95, this study demonstrates the accuracy and reliability of incorporating such tools into clinical practice and decision-making [8].

The PRAEVAorta is an AI software that has enabled fully automatic volumetric segmentation and measurement of infrarenal AAA sac, which is very important for pre-endovascular aortic repair (EVAR) assessment and post-EVAR surveillance. Caradu et al. demonstrated that the software was able to achieve a DSC of 0.95 ± 0.01 and 0.950 ± 0.015 for pre- and post-EVAR, respectively, with reference to manual segmentation while also being significantly quicker than the latter [9, 10].

CT-based segmentation has also been important in interventional oncology. For instance, a study done by Li et al. proposed a multi-task DL model for objective response (OR) prediction and tumor segmentation in HCC patients undergoing transarterial chemoembolization (TACE). The model was trained on preoperative contrast-enhanced CT (CECT) and achieved an AUC of 0.871 in OR prediction and a DSC of 73.6% in tumor segmentation [11]. While there have been numerous models trained to either predict OR or segment the liver tumor, this study was unique as it did both simultaneously.

Often interventional radiologists have to keep track of inferior vena cava filters (IVCF) after placement. This is often challenging due to their small size, deep location, and patients forgetting they have one. Gomes et al. developed a UNet-based model for segmentation of IVCF in abdominopelvic axial CT scans in order to combat this issue. After training their model on 372 CT slices, they achieved a DSC of 0.82 [12].

#### Detection Tasks

A key area of focus in conjunction with EVAR is post-operative endoleak detection. Surveillance for such complications is vital in order to prevent aneurysm growth and sac rupture. Studies have looked into the feasibility of CT images for detecting such endoleaks. Radiomic features extracted from unenhanced post-EVAR CT after aneurysmal sac segmentation have been used to train ML models to detect endoleaks. This has been a successful approach with an average accuracy and AUC of 81% ± 4 and 0.86 ± 0.05, respectively [13]. Another study trained and tested a U-net CNN on CT angiography (CTA) after aneurysm segmentation and data augmentation. Their model achieved an accuracy of 95%, precision of 90%, and recall of 100% compared to subspecialist interpretation [14].

The IVCF segmentation model developed by Gomes et al. as mentioned above was further incorporated into a prediction algorithm that detected and flagged CT scans with IVCFs with an accuracy of 92.22% [12].

#### Prediction and Prognostication Tasks

Studies in this section mainly focused on preoperative imaging to predict postoperative outcomes. Several studies have leveraged CT data and machine learning models to predict outcomes and complications after EVAR. While surveillance imaging is key for follow-up, it becomes less and less frequent as time passes by. Therefore, identifying patients at heightened risk of complication can enable more intense follow-up for those. Similar to detection tasks, radiomic features can also be leveraged for prognostication purposes. Wang et al. for example extracted radiomic features from preoperative CTAs, selected the most significant features and used those to train different ML models for predicting outcomes after EVAR. The logistic regression (LR) model performed the best with an AUC of 0.93 and F1 score of 0.91 [15]. VascAI©, a deep CNN, was developed using preoperative 3D CT images as input to predict complications like endoleak, AAA rupture and graft or vessel occlusions after EVAR. With a sensitivity of 100% and without requiring any expert-level annotations of the input images, the model proves useful potential in quickly identifying patients at risk of postoperative complications [16]. Features extracted from texture analysis of the aneurysm have also been used to train AI classifiers with high accuracy. Ding et al. employed a multilayer perceptron neural network to predict late expansion of AAA after EVAR using early post-EVAR arterial phase-derived aneurysm texture analysis as the input. Achieving an accuracy of 85.17% and an AUC of 0.90 with the gray-level co-occurrence matrix (GLCM) features, this approach proved superior to using clinical and traditional imaging features combined [17]. One successful method has been to extract morphological metrics from images and use those to train AI models. For instance, one study developed an artificial neural network (ANN) to predict the risk of endograft complications and mortality after EVAR. The model used 19 morphological features extracted from CT angiograms using 3Mensio Vascular software. The ANN effectively distinguished between low-risk and high-risk groups, with external validation showing 5-year freedom rates from aortic complications, limb complications, and mortality at 95.9% vs. 67.9%, 99.3% vs. 92.0%, and 87.9% vs. 79.3%, respectively [18]. Predicting sac dimensions in post-EVAR patients who develop type-2 endoleaks is important as early identification of aggressive endoleaks can allow for prompt management. One study used pyradiomics derived radiomic features from CTAs at postoperative months 1 and 6 to predict sac expansion at 1 year with AUCs of 89.3% and 95.5%, respectively. The study used the boruta algorithm for feature selection and SVMs for classifier development [19]. Just like with EVAR, studies have also been done for outcome prediction after TEVAR. A study developed DL models for predicting negative aortic remodelling and risk of reintervention after TEVAR in patients with Stanford type B aortic dissection. Their point cloud neural networks achieved AUCs of 0.884 and 0.836 for both tasks respectively when input was a combination of CTA and clinical features. Since negative remodelling limits long term effectiveness of TEVAR, such models can help identify patients at high post-operative risk and put them in increased imaging surveillance [20].

Automatic segmentation and quantification of vascular calcification has also been used as a measure of reintervention risk after endovascular repair of aorto-iliac occlusive disease. Using CTA as input, one study reported a significantly higher calcification volume in the right and left iliac arteries measured by the algorithm in patients undergoing reintervention [21].

Predicting response to transarterial chemoembolization (TACE) and radioembolization (TARE) has been another area where CT imaging has been used for AI with many of these studies utilizing radiomic features just like with TIPS. Studies have used the pyradiomics package of python for extracting features which are then used to train the models. One study utilized pre-treatment cone-beam CT for input to predict treatment response to TARE of liver metastases, achieving an AUC of 0.85 and a sensitivity of 94.2% [22]. Peng et al. developed a pyradiomics signature for predicting initial treatment response and prognosis during TACE in HCC, achieving AUCs of 0.917, 0.902, and 0.911 in the discovery, validation set 1, and validation set 2, respectively [23]. Another study by a similar group of authors integrated radiomics and DL for predicting the initial treatment response to TACE in HCC, with the random forest algorithm achieving an AUC of 0.967 and DL achieving an AUC of 0.981 in the training cohort [24]. A different approach for treatment prediction was done by another study on TARE. This study improved upon the voxel-based dosimetry model by using an ensemble of deep CNNs for predicting complete treatment response TARE segmentectomy. The model achieved a higher F1-score (0.72 vs. 0.2), accuracy (0.65 vs. 0.60), and sensitivity (1.0 vs. 0.11) compared to the voxel based dosimetry model [25].

Another area of focus has been the use of AI in the prediction of hepatic encephalopathy after TIPS and comparing such models to traditional clinical parameter-based models. Studies have utilized radiomic features extracted from contrast-enhanced abdominal CT images and used those to train traditional ML models for predicting hepatic encephalopathy. Achieving AUCs above 0.8 and outperforming clinical models (AUC = 0.649), this shows their potential in efficient patient selection and clinical decision-making [26, 27]. In conjunction with this has been the prediction of portal hypertension recurrence after TIPS. Using a combination of clinical, demographic, and radiological features derived from postoperative abdominal CT scans, a study developed a scoring system using an ML algorithm based on random forest methodology. The scoring system included a new imaging biomarker called the TIPS-distal end positioning, thereby highlighting the significance of shunt position in disease-free survival [28].

#### Miscellaneous Tasks

Motion compensation and registration of CT imaging with other modalities in the context of VIR procedures has been an area of focus. For instance, CBCT, which is used in IR for complex vascular anatomy, has a long acquisition time and is prone to soft tissue deformable motion. Vessel-targeted autofocus technique, which begins with vasculature identification using a DL model trained on complete sequences of projections followed by an autofocus function, results in improved visibility of fine vascular anatomy that can potentially improve intra-procedural tracking [29]. Studies have used DL to register intraprocedural CBCT with preoperative MR for guidance during TACE procedures. APA2Seg-Net, for instance, generates liver segmentation on the imaging modalities without learning on ground truth, followed by anatomy-guided fusion leading to high-quality multimodal registration [30, 31].

### Magnetic Resonance Imaging (MRI)-Based Studies

#### Prediction and Prognostication Tasks

Studies leveraging AI in MRI imaging have mainly focused on treatment outcomes and prognosis. One study used a DL ResNet model on preoperative MR imaging of uterine fibroids to predict the outcome of uterine fibroid embolization in 409 patients with 727 fibroids. The T1C-trained model outperformed radiologists, achieving a test accuracy of 0.847 compared to the radiologists’ average accuracy of 0.722 [32]. Just like with other procedures, this approach can help with clinician decision-making and early identification of patients with poorer outcomes after fibroid embolization.

Studies have also employed AI in MR imaging for chemo- and radio-embolization of HCC. Two studies by similar groups of authors specifically developed ML models using MR radiomic features in combination with clinical features to predict response to TACE and TARE, respectively. They achieved AUCs of 0.92 and 0.94, respectively, outperforming clinical feature-only and radiomic-only models [33, 34]. Similarly, another study also developed a clinicoradiomic ML model for early prediction of treatment response after TARE, achieving an AUC of 0.89 and again outperforming clinical feature-only and radiomic-only models [35].

### Fluoroscopy and Digital Subtraction Angiography (DSA)-Based Studies

#### Segmentation Tasks

As fluoroscopy and DSA are commonly employed during various VIR procedures, efforts have been made to use these as input for intra-procedural segmentation and localization of devices like guidewires, catheters, and stents. Phantom models and simulations have been commonly used for this purpose. A pipeline using DL was developed and tested that can reconstruct and segment interventional tools like stents and guidewires to provide 4D spatiotemporal information at a similar radiation dose to conventional 2D fluoroscopy. The reported precisions were at least 90%, which makes this approach very promising for tomographic intraprocedural guidance [36]. Similarly, cryo-balloon catheters that are used during pulmonary vein isolation procedures have been automatically segmented and returned as 3D reconstruction using 2 different views, achieving an average localization accuracy of 1.52 pixels or 0.56 mm. This again can provide improved navigation during transcatheter endovascular procedures [37]. Another approach showed how synthetic data and transfer learning can be used to train a CNN to perform robust catheter segmentation in fluoroscopy images, achieving a DSC of ~ 0.55. This point highlights how a robust segmentation model can be made while bypassing the bottleneck of large volumes of manual annotations [38]. Using vessel roadmaps for endovascular navigation has required guidewire segmentation in mask subtraction images. Work has been done to employ DL for guidewire segmentation in non-subtracted X-ray images. This approach outperformed the former mask-subtraction-based technique, with a Dice equal to 58.1% versus 23.7%, respectively [39].

Lastly, efforts have been made to segment the stent placed in the aorta during EVAR. Such segmentations can allow for assessment of fusion of intraoperative X-rays with preoperative information in order to reduce radiation exposure and contrast agent use. This was done by training a CNN model on single uncontrasted X-ray images obtained during the procedures. The model obtained a baseline DSC of 0.933 and an AUC of 0.996 [40]. Automated localization of the stent can help guide optimal deployment intraprocedurally and avoid complications like renal artery occlusion. For this purpose, a study developed a DL model to identify a “No-Go” landing zone during EVAR, defined by the coverage of the lowest renal artery by the stent graft. The model achieved an accuracy and specificity of 0.97 and 0.99, respectively [41].

#### Detection Tasks

Just like with segmentation tasks, detection tasks using fluoroscopy and DSA also focus on the intraprocedural aspect. The InterNet system was developed as a two-stage DL model for detecting active abdominal arterial bleeding using emergency DSA imaging. The doctor's AUC for detecting bleeds improved from 0.759 to 0.803 upon using the model, and the interpretation time significantly decreased from 84.88 to 43.78 s [42].

#### Prediction and Prognostication Tasks

DSA has also been employed as input for prognosis in patients receiving TACE just like CT and MR. A study developed a DL-based framework called DSA-Net for real-time automatic prediction of treatment response to TACE in intermediate-stage HCC patients based on DSA videos. The framework began with a DL-based tumor segmentation model followed by a DL model for response prediction. They achieved an accuracy of 78.2% and 75.1% on internal and external validation cohorts, respectively [43].

#### Miscellaneous Tasks

Since radiation exposure is a notable concern in VIR, an important milestone was presented by the following study. Tang et al. developed a DL approach to reduce radiation in interventional radiology by lowering the acquisition frame rate and using a neural network to interpolate the missing frames, achieving promising results. The average peak signal-to-noise ratio (PSNR) values were 44.94 dB for angiographic, 34.84 dB for subtraction, and 33.82 dB for fluoroscopic modes, with corresponding structural similarity index (SSIM) values of 0.9840, 0.9194, and 0.7752 [44].

### Ultrasound

#### Detection Tasks

US (including duplex scan) is widely used as a screening tool because of its quick nature and no radiation exposure. For this reason, it also has significant applications in detection tasks. One of the common tasks duplex scan is used for its detection of vessel stenosis. One study, for instance, used Doppler waveforms in duplex scans sampled at the ankle vessels as input for a ML algorithm to detect peripheral artery disease. Their highest-performing logistic regression model achieved an AUC of 0.93 [45]. Similarly, another study used the spectral waveforms in duplex scans recorded from the renal arteries to detect significant renal artery stenosis that warranted interventional treatment in the form of angioplasty and stent placement. They employed a 1D-CNN and achieved an AUC of 0.97, again demonstrating the applicability of US as input for AI in regard to VIR [46]. Using a composite input of the ultrasound scan image, spectral waveform, and clinical data has also allowed for the accurate detection of renal artery stenosis using DL models. With an accuracy of 78.25% ± 1.62%, another study reinforced the promising role of such models in assisting critical diagnoses in VIR [47].

Iliac vein compression syndrome is a disease encountered in VIR. Accurate detection of the disease and venous compression points is essential for appropriate clinical management. Work has been done for this purpose by using ultrasound imaging and demonstrated significant reduction in time for diagnosis compared to manual delineation of the disease on angiography images [48].

Moreover, ultrasound and AI have also been used for the detection and evaluation of drug-coated balloon therapy for atherosclerotic obliterans. Using a DL target detection algorithm, this method achieved performance and results better than those of conventional ultrasound technology and on par with DSA, signifying the enhancement AI brings into an imaging modality [49].

### X-Ray

#### Detection Tasks

Plain radiographs have commonly been used as input for detection purposes of different VIR devices because they are widely taken, leading to large datasets. Multiple studies have developed DL models for the detection of the tip of central lines, like central venous catheters and peripherally inserted central catheters. The tip position is prevalently confirmed after line placement on X-rays since malposition can lead to various complications, like punctures, embolisms, and arrhythmias. For instance, Lee et al. proposed an AI system of 2 CNNs for detecting PICC line and tip location with a mean absolute distance from ground truth of 3.10 mm (± 2.03 mm), and a root mean square error (RMSE) of 3.71 mm [50]. The performances of these models highlight their applicability in the clinical workflow for automated detection of catheter tips in order to avoid complications and improve the efficiency of radiologists [50–53].

Along with the detection of IVC filters, as discussed previously, it is also important to know their type, which is essential when monitoring for complications and overall patient management. Ni et al. employed a CNN model that used plain radiographic images as input for detecting 14 different types of IVC filters. The model achieved an F1 score of 0.97 and highlighted salient features it used for the task in the form of heatmaps. This shows promise in the application of such tools in optimizing patient care for those with indwelling devices like filters and pacemakers [54].

### Multimodality and Hybrid Studies

#### Prediction and Prognostication Tasks

Efforts of combining input from multiple imaging modalities for AI-based work have been made previously. Combination of imaging variables from DSA and CTA together with clinical variables has been used to construct ML models for risk prediction after TEVAR for type B aortic dissection. In the study, the LR model performed the best in predicting distal aortic enlargement and ANN for distal aneurysm formation with AUCs of 0.773 and 0.876, respectively [55].

## Conclusion and Future Directions

The application of artificial intelligence (AI) in vascular interventional radiology (VIR) has gained significant traction in recent years, as evidenced by the numerous studies included in this review. The diverse array of imaging modalities and tasks addressed by AI models underscores their versatility and potential impact in enhancing diagnostic accuracy, treatment planning, and patient outcomes.

One of the notable strengths of AI approaches in VIR is their ability to perform precise and automated segmentation of anatomical structures and interventional devices. DL models, particularly CNNs, have demonstrated exceptional performance in segmenting AAA sacs, endoleaks, blood vessels, and interventional tools from various imaging modalities, including computed tomography (CT), magnetic resonance imaging (MRI), fluoroscopy, and digital subtraction angiography (DSA). The high Dice similarity coefficients (DSCs) and Jaccard indices achieved by these models, often surpassing human performance, highlight their potential for accurate and efficient segmentation tasks crucial for diagnosis, treatment planning, and post-procedural assessment.

Another significant application of AI in VIR is the detection of critical structures and events, such as active bleeding, stent deployment, and arteriovenous fistula stenosis. Studies using DL models like InterNet have shown promising results in detecting active abdominal arterial bleeding from DSA images [42].

**Fig. 2:**
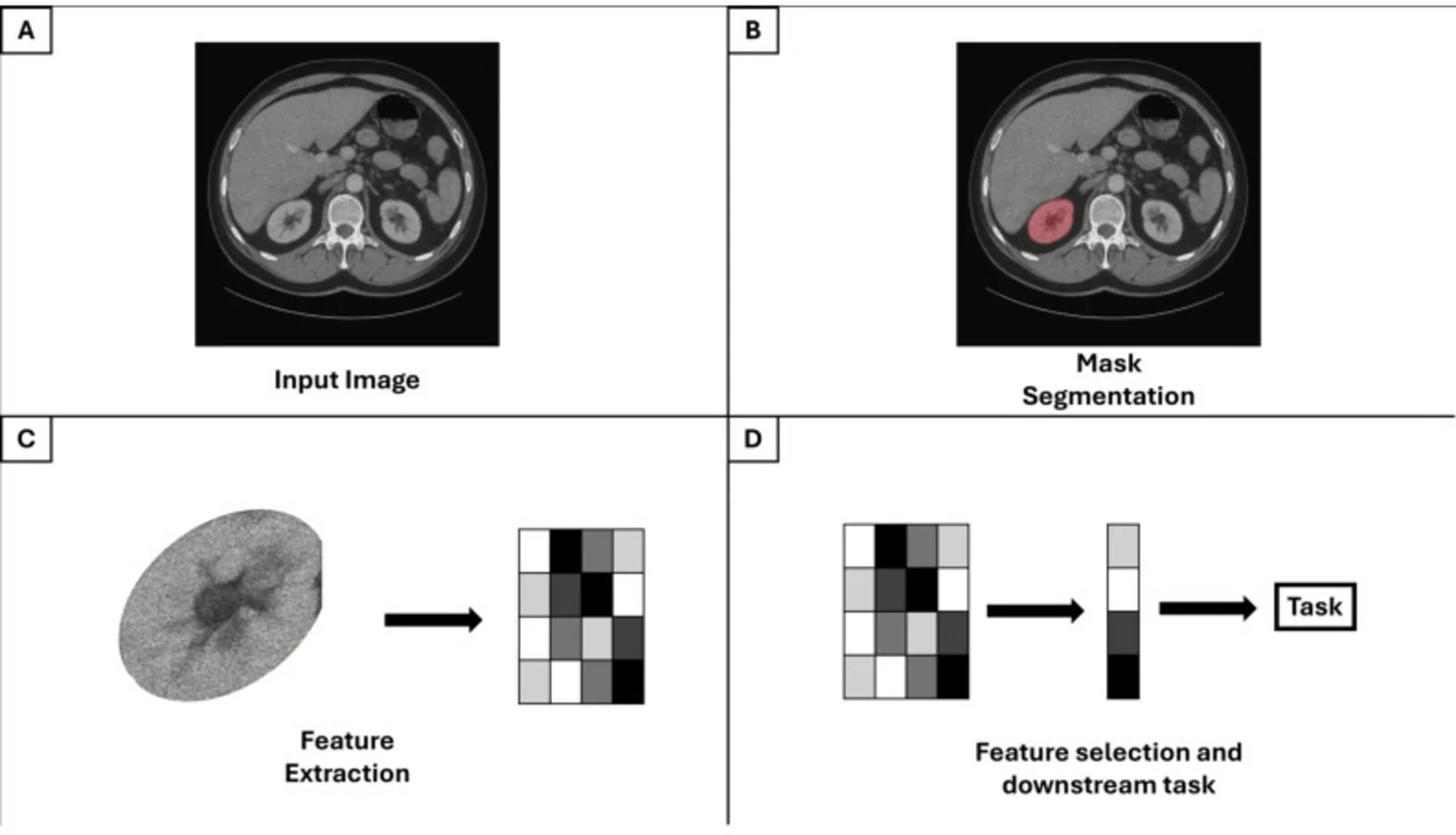
Illustration schema of an input image (**A**) undergoing region-of-interest segmentation (**B**) followed by feature extraction (**C**), feature selection, and then downstream task execution (**D**)

Predicting treatment response, complications, and disease progression is another area where AI has shown substantial promise in VIR. Radiomics-based approaches, which extract quantitative features from medical images, have been widely employed in combination with machine learning algorithms to predict outcomes after interventions like EVAR, TACE, TARE, and TIPS. Figure 2 illustrates a common approach seen in such models where the input images first undergo segmentation of the region of interest followed by feature extraction; the extracted features then normally undergo selection before being used for downstream task execution like in Wang et al. [15]. Studies like those by Wang et al., Peng et al., and Cheng et al. have demonstrated the ability of these models to accurately predict EVAR-related adverse events, treatment response to TACE and TARE, and disease-free survival after TIPS, often outperforming traditional clinical and radiological assessment methods [15, 23, 24, 26, 27]. These predictive capabilities can inform personalized treatment strategies, risk stratification, and post-procedural surveillance, ultimately improving patient care and resource allocation.

The clinical deployment of AI in VIR also necessitates careful consideration of regulatory and ethical frameworks. In the United States, AI-based medical devices must navigate FDA approval pathways, typically classified as Software as a Medical Device (SaMD), with the regulatory burden varying based on the intended use and risk level of the application. The FDA’s evolving framework for adaptive AI algorithms presents unique challenges for models that continuously learn from new data, requiring robust post-market surveillance and predetermined change control plans. Beyond regulatory approval, ethical considerations around data privacy and security are paramount, particularly given the sensitive nature of medical imaging data and patient information. Compliance with regulations such as HIPAA in the US and GDPR in Europe is essential, necessitating secure data storage, de-identification protocols, and transparent consent processes. Furthermore, issues of algorithmic bias, equitable access to AI-enhanced care, and liability in cases of AI-assisted diagnostic or treatment errors remain areas requiring ongoing dialogue between clinicians, AI developers, regulatory bodies, and ethicists. Establishing clear guidelines for data governance, model validation, and clinical responsibility will be critical for the safe and equitable integration of AI technologies into interventional radiology practice.

Despite the promising results and potential benefits, several challenges and limitations exist in the application of AI in VIR. One major challenge is the limited availability of large, high-quality, and diverse datasets for the training and validation of AI models [56]. Many studies in this review relied on relatively small sample sizes or single-center data, which may limit the generalizability and robustness of the developed models [57]. Additionally, the inherent complexity and variability of interventional procedures, patient populations, and imaging protocols can pose challenges for AI model development and deployment. Another limitation is the potential for bias and overfitting in AI models, particularly when trained on datasets with imbalanced or skewed distributions [58]. This can lead to suboptimal performance or inaccurate predictions in underrepresented subgroups or rare clinical scenarios. Rigorous validation, external testing, and continuous model refinement are necessary to mitigate these risks and ensure the reliability and fairness of AI-based solutions.

Furthermore, the interpretability and transparency of complex AI models, particularly DL architectures, remain a challenge. While these models can achieve high-performance metrics, their inner workings and decision-making processes are often opaque, making it difficult for clinicians to understand and trust the model’s outputs. Efforts to develop explainable AI (xAI) techniques and integrate human expertise into the decision-making process are crucial for fostering trust and ensuring the responsible deployment of AI in clinical settings [59].

Despite these challenges, the potential benefits of AI in VIR are significant and warrant continued research and development efforts. The integration of AI technologies into interventional radiology workflows can streamline and enhance various aspects of patient care, from diagnostic accuracy and treatment planning to procedural guidance and post-procedural monitoring [60–62].

One promising area for future research is the development of real-time, intraoperative AI-assisted systems that can provide continuous guidance and decision support during interventional procedures. By leveraging the rapid processing capabilities of AI models and integrating them with interventional imaging systems, these solutions can offer real-time feedback on device positioning, anatomical variations, and procedural risks, potentially reducing procedural times, radiation exposure, and complications.

Another area of potential exploration is the integration of AI with augmented reality (AR) and virtual reality (VR) technologies for interventional radiology training and simulation. AI-powered virtual environments can provide realistic simulations of interventional procedures, enabling trainees to practice and refine their skills in a safe and controlled setting. Additionally, AR-based guidance systems can overlay AI-generated annotations and visualizations onto real-time imaging data, enhancing spatial awareness and procedural accuracy for interventional radiologists.

In conclusion, the application of AI in vascular interventional radiology has the potential to revolutionize various aspects of patient care, from diagnostic accuracy and treatment planning to procedural guidance and post-procedural monitoring. While challenges and limitations exist, the promising results demonstrated by the studies included in this review highlight the versatility and potential impact of AI technologies in this field. Continued research, collaboration between clinicians and AI experts, and responsible deployment of AI solutions are crucial for realizing the full potential of these technologies and improving patient outcomes in interventional radiology.

## Supplementary Information

Below is the link to the electronic supplementary material.

ESM 1 (DOCX 547 KB)
