# Supplementary material for: Imaging-Based Artificial Intelligence in Vascular and Interventional Radiology: A Narrative Review
Source: J Imaging Inform Med. 2026 Jan 5;39(5):3810–9. doi: 10.1007/s10278-025-01803-0 (PMC13694673; doi:10.1007/s10278-025-01803-0)
Supplement: Supplementary file 1 [file 10278_2025_1803_MOESM1_ESM.docx]

**Section 1: Search terms**

**PubMed:**

“Artificial Intelligence”[Mesh], "Neural Networks, Computer"[Mesh], "Deep Learning"[Mesh], "Machine Learning"[Mesh], "Supervised Machine Learning"[Mesh], "Unsupervised Machine Learning"[Mesh], "artificial intelligence", "neural network", "machine learning", "deep learning", "unsupervised learning", "unsupervised machine learning", "supervised machine learning", "supervised learning", "unsupervised deep learning", "supervised deep learning", "Angiography, Digital Subtraction"[Mesh], "Radiology, Interventional"[Mesh], "Radiography, Interventional"[Mesh], "Embolectomy"[Mesh], "Endovascular Procedures"[Mesh], "Portasystemic Shunt, Transjugular Intrahepatic"[Mesh], "Limb Salvage"[Mesh], "interventional radiology", "image guided intervention", "minimally invasive intervention", "image guided therapy", "image guided surgery", "digital subtraction", "endovascular procedure", "embolectomy", "portasystemic shunt transjugular intrahepatic", "limb salvage", "angioscopy".

**Embase:**

"artificial intelligence", "neural network", "machine learning", "deep learning", "unsupervised learning", "unsupervised machine learning", "supervised machine learning", "supervised learning", "unsupervised deep learning", "supervised deep learning", "interventional radiology", "image guided intervention", "minimally invasive intervention", "image guided therapy", "image guided surgery", "digital subtraction", "endovascular procedure", "embolectomy", "portasystemic shunt transjugular intrahepatic", "limb salvage", "angioscopy".

**Web of Science:**

"artificial intelligence", "neural network", "machine learning", "deep learning", "unsupervised learning", "unsupervised machine learning", "supervised machine learning", "supervised learning", "unsupervised deep learning", "supervised deep learning", "interventional radiology", "image guided intervention", "minimally invasive intervention", "image guided therapy", "image guided surgery", "digital subtraction", "endovascular procedure", "embolectomy", "portasystemic shunt transjugular intrahepatic", "limb salvage", "angioscopy".

**Section 2: Tabulation of literature review**

The following tables group the included studies and their models based on the input modality.

**Table E1: CT based studies:**

| Year | Title | Author | Procedure (if any) | Dataset | Description | Metric used |
| --- | --- | --- | --- | --- | --- | --- |
| Segmentation | | | | | | |
| 2023 | Deep learning-based multi-stage postoperative type-b aortic dissection segmentation using global-local fusion learning | Zhang et al. (7) | EVAR | 306 scan images | Segmentations of post-operative type-B dissection after EVAR | Dice Score Coefficient |
| 2022 | A Deep Learning-Based and Fully Automated Pipeline for Thoracic Aorta Geometric Analysis and Planning for Endovascular Repair from Computed Tomography | Saitta et al. (8) | TEVAR | 465 CT scans | Fully automated pipeline to identify geometric landing zones for endograft deployment during TEVAR | Mean Dice Score |
| 2021 | Fully automatic volume segmentation of infrarenal abdominal aortic aneurysm computed tomography images with deep learning approaches versus physician controlled manual segmentation | Caradu et al. (9) | EVAR | 100 CT scans | PRAEVAorta used to segment infrarenal AAA | Mean Dice Similarity Coefficient, Jaccard Index, Sensitivity, Specificity, Mean Hausdorff distance |
| 2022 | Fully automatic volume segmentation using deep learning approaches to assess aneurysmal sac evolution after infrarenal endovascular aortic repair | Caradu et al. (10) | EVAR | 48 patients | PRAEVAorta used to segment post-Op AAA sac evolution after EVAR | Mean Dice Similarity Coefficient, Jaccard Index, Sensitivity, Specificity, Mean Hausdorff distance, Volumetric similarity |
| 2022 | Multi-Task Deep Learning Approach for Simultaneous Objective Response Prediction and Tumor Segmentation in HCC Patients with Transarterial Chemoembolization | Li et al. (11) | TACE | 248 patients | Deep learning model to simultaneously predict objective response and tumor segmentation in HCC patients who underwent TACE | AUC, Dice Coefficient |
| Detection | | | | | | |
| 2022 | Application of Deep Learning to IVC Filter Detection from CT Scans | Gomes et al. (12) | IVC filter | 465 CT image slices from 90 CT scans | Detecting and segmenting IVC filter in CT scan slices | Dice Score, Accuracy |
| 2023 | Unenhanced computed tomography radiomics help detect endoleaks after endovascular repair of abdominal aortic aneurysm. | Hu et al. (13) | EVAR | 216 patients | Detecting endoleak in unenhanced Ct scans of AAA after EVAR | AUC, Accuracy, Sensitivity, Specificity |
| 2020 | Machine learning for endoleak detection after endovascular aortic repair | Talebi et al. (14) | EVAR | 70 patients | Detecting AAA endoleak after EVAR | Accuracy, Precision, Recall |
| Prediction and prognostication | | | | | | |
| 2023 | A radiomics model for predicting the outcome of endovascular abdominal aortic aneurysm repair based on machine learning. | Wang et al. (15) | EVAR | 493 patients; 30 radiomic features extracted from each | Predicting outcome and severe adverse effects after EVAR | AUC, Accuracy, F1-score, Rad-score waterfall plot, Calibration curve, Decision curve analysis |
| 2024 | Applying Artificial Intelligence to Predict Complications After Endovascular Aneurysm Repair | Long et al. (16) | EVAR | 273 patients | Predicting patients that will have complications following EVAR | Sensitivity |
| 2020 | CT texture analysis predicts abdominal aortic aneurysm post-endovascular aortic aneurysm repair progression | Ding et al. (17) | EVAR | 99 patients | Predicting aneurysm expansion using grey-level matrices extracted Ct texture features from the first postoperative CT | AUC, Accuracy |
| 2015 | An Artificial Neural Network Stratifies the Risks of Reintervention and Mortality after Endovascular Aneurysm Repair; a Retrospective Observational study | Karthikesalingam at al. (18) | EVAR | 761 patients | Predicting mortality and need for reintervention following EVAR using 19 morphological CT features | Kaplan-Meier analysis |
| 2022 | Radiomics and machine learning to predict aggressive type 2 endoleaks after endovascular aneurysm repair: a proof of concept | Charalambous et al. (19) | EVAR | 45 CTAs from 15 patients | Predicting the risk of aggressive type 2 endoleaks after EVAR | AUC, Specificity, Sensitivity |
| 2024 | Deep Learning Prediction for Distal Aortic Remodeling After Thoracic Endovascular Aortic Repair in Stanford Type B Aortic Dissection | Zhou et al. (20) | TEVAR | 147 patients | Prediction for distal aortic remodelling after TEVAR in patients of Stanford type B aortic dissection | AUC |
| 2022 | Automatic Measurement of Vascular Calcifications in Patients with Aorto-Iliac Occlusive Disease to Predict the Risk of Re-intervention After Endovascular Repair | Guidi et al. (21) | Endovascular repair for aortoiliac occlusive disease | 171 patients | Automatic quantification of vascular calcifications to predict response to endovascular repair and risk of re-intervention | Calcification volume comparison between re-intervention and non-reintervention group |
| 2021 | Prediction of treatment response to transarterial radioembolization of liver metastases: Radiomics analysis of pre-treatment cone-beam CT: A proof of concept study | Kobe et al. (22) | TARE | 104 patients | Using radiomic features to predict response to TARE in hepatocellular carcinoma patients | AUC, Sensitivity, Specificity |
| 2021 | Development and validation of a pyradiomics signature to predict initial treatment response and prognosis during transarterial chemoembolization in hepatocellular carcinoma | Peng et al. (23) | TACE | 139 patients | Predicting initial response to TACE using radiomic features and deep learning | AUC |
| 2022 | Predicting the Initial Treatment Response to Transarterial Chemoembolization in Intermediate-Stage Hepatocellular Carcinoma by the Integration of Radiomics and Deep Learning. | Peng et al. (24) | TACE | 313 patients | Predicting initial response to TACE using radiomic features and deep learning | AUC |
| 2023 | Using Deep Learning to Predict Treatment Response in Patients with Hepatocellular Carcinoma Treated with Y90 Radiation Segmentectomy. | Wagstaff et al. (25) | TARE | 77 patients | Predict treatment response following TARE in HCC patients and potentially identify treatment failure patients that may need additional follow-up and treatment | FI score, Accuracy, Sensitivity |
| 2022 | CT-based radiomics model for preoperative prediction of hepatic encephalopathy after transjugular intrahepatic portosystemic shunt. | Cheng et al. (26) | TIPS | 106 patients | Predicting hepatic encephalopathy after TIPS using radiomic features and random forest algorithm | AUC |
| 2024 | Prediction of Hepatic Encephalopathy After Transjugular Intrahepatic Portosystemic Shunt Based on CT Radiomic Features of Visceral Adipose Tissue. | Cheng et al. (27) | TIPS | 130 patients | Predicting hepatic encephalopathy after TIPS using CT radiomic features of visceral adipose tissue | AUC, Sensitivity, Specificity, Positive predictive value, Negative predictive value |
| 2022 | Predicting death or recurrence of portal hypertension symptoms after TIPS procedures. | Sun et al (28) | TIPS | 206 patients each with 7 epidemiological, 4 clinical and 9 radiological variables | Signature prediction model for disease-free survival after TIPS procedure in cirrhotic patients with portal hypertension | Hazard ratio |
| Registration and fusion | | | | | | |
| 2023 | Deformable Motion Compensation for Intraprocedural Vascular Cone-beam CT with Sequential Projection Domain Targeting and Vessel-Enhancing Autofocus. | Lu et al. (29) | N/A | Training and validation= CT-ORG dataset; Hold out test set= 224 sequences | Automated motion compensation via targeted autofocus to improve visibility of vascular anatomy in abdominal CBCT | Intersection-over-union, 3D Dice score |
| 2021 | Anatomy-guided multimodal registration by learning segmentation without ground truth: Application to intraprocedural CBCT/MR liver segmentation and registration | Zhou et al. (30) | TACE | 16 patients | Anatomy guided intraprocedural CBCT/MRI liver segmentation and registration without ground truth | Dice Similarity Coefficient, Average Symmetric Surface Distance |
| 2021 | Automatic Multimodal Registration via Intraprocedural Cone-Beam CT Segmentation using MRI Distance Maps. | Augenfeld at al. (31) | TACE | 16 patients | Fully automated framework to register diagnostic MRI to intraprocedural CBCT to guide TACE procedure | Dice similarity coefficient, AUC |

**Table E2: MRI based studies:**

| Year |  | Author | Procedure (if any) | Dataset | Description |  |
| --- | --- | --- | --- | --- | --- | --- |
| Prediction and prognostication | | | | | | |
| 2020 | Deep Learning Based on MR Imaging for Predicting Outcome of Uterine Fibroid Embolization | Luo et al. (32) | UAE | 409 patients | Deep learning model on uterine fibroids to predict outcome following UAE | Accuracy, Sensitivity, Specificity |
| 2023 | Machine Learning Models in Prediction of Treatment Response After Chemoembolization with MRI Clinicoradiomics Features. | Ince et al. (33) | TACE | 188 patients | Machine learning models based on clinio-radiomic features showed potential to predict response to TACE | AUC |
| 2023 | Prediction of Response of Hepatocellular Carcinoma to Radioembolization: Machine Learning Using Preprocedural Clinical Factors and MR Imaging Radiomics. | Ince et al. (34) | TARE | 82 patients | Prediction of response to TAE for hepatocellular carcinoma using laboratory, clinical, procedural and radiomic features | AUC |
| 2023 | Early Prediction of Response of Hepatocellular Carcinoma to Yttrium-90 Radiation Segmentectomy Using a Machine Learning MR Imaging Radiomic Approach | Marinelli et al. (35) | TARE | 76 patients | Predicting response to TARE from radiomic features extracted from baseline and follow-up MR imaging | AUC |

**Table E3: Fluoroscopy and DSA based studies:**

| Year |  | Author | Procedure (if any) | Dataset | Description |  |
| --- | --- | --- | --- | --- | --- | --- |
| Segmentation | | | | | | |
| 2021 | Deep learning‐based reconstruction of interventional tools and devices from four X‐ray projections for tomographic interventional guidance | Eulig et al. (36) | Tools and devices |  | Two-stage deep learning approach for real-time tomographic guidance of interventional tools | Precision |
| 2021 | Cryo-balloon catheter localization in X-Ray fluoroscopy using U-net | Vernikouskaya at el. (37) | Pulmonary vein isolation procedure | 15 patients | Deep learning approach to identify cryo-balloon marker and catheter shaft in 2D fluoroscopy during pulmonary vein isolation | Prediction time per frame, Localization accuracy |
| 2020 | Catheter segmentation in X-ray fluoroscopy using synthetic data and transfer learning with light U-nets. | Gherardini et al. (38) | Tools and devices | 3 datasets with 9000, 2000, and 1207 images. | Realtime catheter and wire segmentation in fluoroscopy with deep learning | Dice coefficient |
| 2019 | Deep learning based guidewire segmentation in x-ray images. | Wagner et al. (39) | Liver embolization | Trained on a set of 56768 images from 10 acquisitions | Segmentation of guidewire in Xray fluoroscopy during liver embolization procedures | Dice, False negative rate |
| 2018 | Intraoperative stent segmentation in X-ray fluoroscopy for endovascular aortic repair | Breininger et (40) al. | EVAR | 63 patients | Detection and segmentation of aortic stent in uncontrasted chest Xray | Dice coefficient, AUC |
| Detection | | | | | | |
| 2024 | Development of an Artificial Intelligence Tool for Intraoperative Guidance During Endovascular Abdominal Aortic Aneurysm Repair. | Li et al. (41) | EVAR | 369 images from 110 patients/videos | Model for identifying ‘No-Go’ landing zones during EVAR | Intersection-over-union, F1 score, Accuracy, Sensitivity, Specificity, Positive Predictive value, Negative predictive value. |
| 2022 | InterNet: Detection of Active Abdominal Arterial Bleeding Using Emergency Digital Subtraction Angiography Imaging With Two-Stage Deep Learning | Min et al. (42) | Abdominal arterial bleed detection | 660 patients | Two-stage model for detecting active abdominal bleed in patients undergoing abdominal DSA procedures | Average precision, precision, recall, AUC, elapsed time for evaluation |
| Prediction and prognostication | | | | | | |
| 2022 | Real-time automatic prediction of treatment response to transcatheter arterial chemoembolization in patients with hepatocellular carcinoma using deep learning based on digital subtraction angiography videos. | Zhang et al. (43) | TACE | 605 patients | Model for automatic real-time prediction of response to TACE treatment from DSA videos | Dice co-efficient, AUC |
| Motion compensation and frame interpolation | | | | | | |
| 2024 | Radiation reduction for interventional radiology imaging: a video frame interpolation solution | Tang et al. (44) |  | 1367 sequences | Model for intraprocedural video frame interpolation to reduce radiation exposure during procedure | Peak signal-to-noise ratio, structural similarity index |

**Table E4: US based studies:**

| Year |  | Author | Procedure or disease (if any) | Dataset | Description |  |
| --- | --- | --- | --- | --- | --- | --- |
| Prediction and prognostication | | | | | | |
| 2022 | Machine learning-based classification of arterial spectral waveforms for the diagnosis of peripheral artery disease in the context of diabetes: A proof-of-concept study. | Normanhi et al. (45) | Peripheral artery disease | 590 spectral waveforms | Model for diagnosing peripheral artery disease using spectral arterial waveforms in duplex ultrasound of ankle vessels | Accuracy, Sensitivity, Specificity, AUC |
| 2025 | Pilot Study of Using Machine Learning to Detect Atherosclerotic Renal Artery Stenosis From Spectral Doppler Waveforms. | Mukhtar et al. (46) | Renal artery stenosis | 396 spectral waveforms from 198 patients | Model for detecting significant renal artery stenosis indicative for interventional treatment | Accuracy, precision, AUC |
| 2024 | Deep-learning-based renal artery stenosis diagnosis via multimodal fusion. | Wang et al. (47) | Renal artery stenosis | 1485 patients | Multimodal fusion model for diagnosing renal artery stenosis | Accuracy, Precision, Recall |
| 2021 | Effect of Interventional Therapy on Iliac Venous Compression Syndrome Evaluated and Diagnosed by Artificial Intelligence Algorithm-Based Ultrasound Images | Bai et al. (48) | May Thurner syndrome treatment | 284 patients | Model for ultrasound image evaluation and analysis during May-Thurner syndrome treatment for detecting lower extremity vein lesions. | Call-up rate, precision, accuracy |
| 2022 | Ultrasonic Image Feature Analysis under Deep Learning Algorithm to Evaluate the Efficacy of Drug-Coated Balloon for Treatment of Arteriosclerotic Occlusion. | Zhang et al. (49) | PAD angioplasty | 56 patients | Model for evaluating DCB efficacy for lower extremity arteriosclerotic disease using ultrasound image feature analysis | Dice, Precision, Sensitivity |

**Table E5: X-ray based studies:**

| Year |  | Author | Procedure (if any) | Dataset | Description |  |
| --- | --- | --- | --- | --- | --- | --- |
| Detection | | | | | | |
| 2018 | A Deep-Learning System for Fully-Automated Peripherally Inserted Central Catheter (PICC) Tip Detection. | Lee et al. (50) | PICC | 450 images | 2 NN models used to detect PICC line and tip location in chest X Rays | Root mean squares error |
| 2023 | Automated precision localization of peripherally inserted central catheter tip through model-agnostic multi-stage networks | Park et al. (51) | PICC | 560 images | Two-stage model to accurately determine the tip location of PICC in chest Xrays | Root mean squared error, Ratio of single fragment images |
| 2023 | High-resolution feature based central venous catheter tip detection network in X-ray images. | Wang et al. (52) | CVC | 1822 images | CNN based model to detect tip of CVC in a public dataset of chest Xrays | Mean pixel error |
| 2020 | Detection of peripherally inserted central catheter (PICC) in chest X-ray images: A multi-task deep learning model | Yu et al. (53) | PICC | 348 images | Model for predicting PICC location in chest Xrays | Precision, Recall, F1 score |
| Detection | | | | | | |
| 2020 | Deep Learning for Automated Classification of Inferior Vena Cava Filter Types on Radiographs. | Ni et al. (54) | IVC filter | 1375 images | CNN based model for automated identification of different IVC filters | F1 score |

**Table E6: Multimodality and hybrid studies:**

| Year |  | Author | Procedure (if any) | Dataset | Description |  |
| --- | --- | --- | --- | --- | --- | --- |
| Prediction and prognostication | | | | | | |
| 2021 | Prediction of Distal Aortic Enlargement after Proximal Repair of Aortic Dissection Using Machine Learning | Zhou et al. (55) | TEVAR | 503 patients each with 38 clinical and imaging variables (extracted from CTA and angiography) | Predicting distal aortic enlargement after TEVAR treatment for type B aortic dissection | AUC, Sensitivity, Specificity |
